# CT and MR Imaging Findings of Pancreatic Paragangliomas: A Case Report

**DOI:** 10.1097/MD.0000000000002959

**Published:** 2016-03-07

**Authors:** Wenjie Liang, Shunliang Xu

**Affiliations:** From the Department of Radiology, The First Affiliated Hospital, College of Medicine, Zhejiang University, Hangzhou, Zhejiang, China.

## Abstract

Previous studies on pancreatic paraganglioma, a rare neoplasm, have primarily reported its ultrasound and routine and contrast-enhanced computed tomography (CT) findings. To our knowledge, we are the first to report the contrast-enhanced magnetic resonance imaging (MRI) and diffusion-weighted imaging (DWI) sequence findings of pancreatic paraganglioma.

A male patient, ages 41 years, was admitted to our hospital due to a pancreatic space-occupying lesion that had been present for more than 10 days. The patient had no obvious discomfort. He had a history of hypertension and hyperthyroidism. Physical examination revealed upper abdominal tenderness without a palpable mass. Routine and contrast-enhanced abdominal CT showed a soft tissue mass at the pancreatic head/uncinate process, with patchy calcification within the lesion. On a contrast-enhanced CT scan, severe enhancement of the mass in the arterial phase was noted, as was slightly reduced but still marked enhancement in the venous phase. The celiac trunk and superior mesenteric artery segment were wrapped by the tumor. Thickened, tortuous vessels were observed at the lesion edges, around which there were multiple enlarged lymph nodes. The main pancreatic duct was markedly dilated. Routine and contrast-enhanced pancreatic MRI demonstrated an abnormal nodular signal in the pancreatic head/uncinate process that was approximately 4.3 × 6.4 cm^2^ in size. T1-weighted imaging (T1WI) revealed hypointensity, whereas T2-weighted imaging (T2WI) revealed nonhomogeneous, slight hyperintensity. Patchy hypointensity on both T1WI and T2WI was observed within the lesion. DWI showed slight hyperintensity. Grossly heterogeneous enhancement of the mass was observed on a contrast-enhanced MRI scan, with the tumor wrapped around the adjacent vasculature, and multiple enlarged lymph nodes were observed peripherally. After preoperative preparation, the patient underwent pancreatoduodenectomy. Histopathology and immunohistochemistry of the resected tumor indicated pancreatic paraganglioma. After surgery, the patient recovered well, without presenting any recurrence or metastasis during short-term follow-up.

For hypervascular pancreatic tumors on contrast-enhanced CT or MRI, and particularly those occurring in the pancreatic head, with a clear display of draining veins, the possibility of pancreatic paraganglioma should be considered. These tumors usually exhibit necrosis or cystic changes and are occasionally accompanied by calcification.

## INTRODUCTION

Paraganglioma is a type of tumor derived from neural crest cells. During the development of paraganglioma, neural crest cells, which are distributed in a scattered pattern in the body, aggregate to form paraganglia. Most paragangliomas associated with the parasympathetic system occur in the cranial base, neck, and anterior mediastinum, including the vagal paraganglia, carotid body paraganglion, and jugulotympanic paraganglia. The most common paragangliomas are carotid body tumors and glomus jugulare tumors; such tumors have a chemoreceptor function.^1^ Paragangliomas associated with the sympathetic system can occur in any part of the sympathetic chain, such as the posterior mediastinum and thoracolumbar paravertebral region, including Zuckerkandl's body; the latter is more common, and these tumors are considered to have functions similar to those in the adrenal medulla.^1^ The adrenal medulla is a special type of paraganglion. Paragangliomas originating in the adrenal medulla have a specific name, pheochromocytomas, whereas those occurring in other parts of the adrenal medulla are called extra-adrenal paragangliomas. Approximately 1-quarter of sporadic pheochromocytoma patients may have related gene mutations, including mutations of the RET gene (associated with multiple endocrine neoplasia type 2), VHL gene (associated with von Hippel–Lindau disease), succinate dehydrogenase subunit B gene (SDHB), and succinate dehydrogenase subunit D (SDHD) gene.^2^ In addition, extra-adrenal paraganglioma patients are considered to have a potential genetic predisposition.^2^ To our knowledge, paragangliomas rarely occur in the pancreas; indeed, there are thus far only 26 cases of pancreatic paragangliomas reported in the English-language literature.^3–22^ Here, we report a case of pancreatic paraganglioma with complete clinical and imaging data. Additionally, we are the first to report its contrast-enhanced magnetic resonance imaging (MRI) and diffusion-weighted imaging (DWI) sequence findings.

## CONSENT

The patient signed the necessary documents to consent to the use of his data for teaching and publication.

## CASE REPORT

A male patient, ages 41 years, was hospitalized due to a pancreatic space-occupying lesion that had been present for more than 10 days. Ten days prior, the patient had gone to a local hospital for medical examination, and a duodenal ampullary mass had been found on abdominal ultrasound examination. Routine and contrast-enhanced pancreatic MRI at the local hospital had shown a space-occupying lesion in the pancreatic head, and the possibility of malignancy with celiac truncal and superior mesenteric arteriovenous involvement had been considered. The patient had no abdominal pain, abdominal distension, nausea, vomiting, chills, or fever at that time and did not receive treatment. At the most recent presentation, the patient had no discomfort, such as chills, fever, dizziness, headache, nausea, or vomiting, and was visiting our hospital for further diagnosis and treatment. The patient had an 8-month history of hypertension, with his highest blood pressure being 210/120 mm Hg. He was taking antihypertensive drugs, such as oral nifedipine and metoprolol, and claimed that his blood pressure was well controlled. The patient had a >10-year history of hyperthyroidism, had previously received I^131^ isotope therapy and was taking oral euthyrox for treatment. He denied a family history of related diseases. On physical examination, the abdomen was flat and soft, and upper abdominal tenderness was noted, without rebound tenderness or a palpable mass. Routine blood tests revealed a slightly elevated neutrophil count (77.0% [normal range: 50.0–70.0%]) and reduced lymphocyte and eosinophil counts (13.4% [normal range: 20.0–40.0%] and 0.3% [normal range: 0.5–5.0%], respectively), whereas the remaining indices were generally within normal ranges. The liver function index, that is, the alanine aminotransferase level, was mildly elevated, to 49 U/L (normal range: 5–40 U/L), whereas the remaining liver, kidney, and electrolyte indices were all generally within normal ranges. The ferritin index had risen to 360.7 ng/mL (normal range: 7.0–323.0 ng/mL), whereas the remaining tumor markers, including α-fetoprotein, carcinoembryonic antigen, carbohydrate antigen 19-9, carbohydrate antigen 125, and total prostate-specific antigen, were all within the normal ranges. Hepatitis B surface antigen, hepatitis C antigen, anti-HIV antibody, and anti-*Treponema pallidum* antibodies were all negative.

**FIGURE 1:**
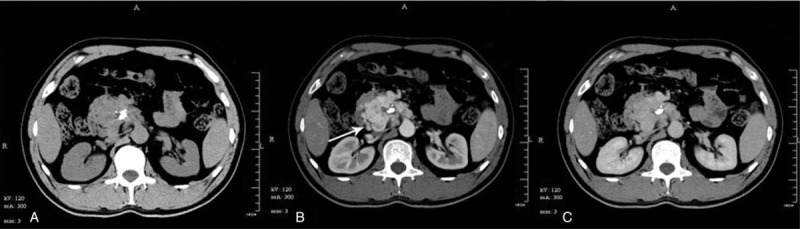
(A) Routine abdominal CT showing an isodense mass in the pancreatic head relative to the liver density, with lobulated margins and the presence of calcification. (B) Contrast-enhanced abdominal CT showing severe heterogeneous enhancement of the mass, with a clear boundary and multiple tortuous blood vessels in the arterial phase observed peripherally (arrow). (C) Slightly decreased venous phase enhancement of the mass, which still appears relatively hyperdense.

**FIGURE 2:**
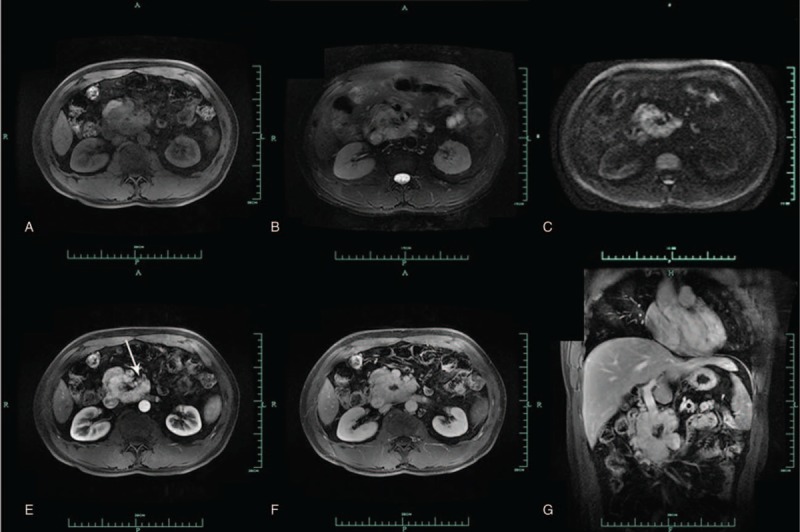
(A) Routine pancreatic MRI showing slightly heterogeneous hypointensity of the pancreatic head mass on T1WI, with a clear boundary. (B) T2WI of the mass, showing heterogeneous, slight hyperintensity. (C) DWI sequence of the mass, showing slight hyperintensity. (D) Contrast-enhanced pancreatic MRI showing severe mass enhancement, with no enhancement of the central focus. Draining vein of the mass in the arterial phase are also observed peripherally (arrow). (E) Slightly decreased venous phase enhancement of the mass, which still shows relative hyperintensity. (F) Coronal enhancement of the mass, tending to be homogeneous and hyperintense, with no central enhancement, and occurring in the delayed phase.

Routine and contrast-enhanced abdominal computed tomography (CT) showed a markedly enlarged pancreatic head/uncinate process, within which extension of an irregular mass to the top of the horizontal duodenal segment along the retroperitoneal space was observed, with an average density of approximately 48.9 Hounsfield units (HU). Patchy calcification was observed within the lesion. On a contrast-enhanced CT scan, heterogeneous enhancement of the mass was noted, with an average arterial phase density of approximately 176.6 HU and an average venous phase density of approximately 106.1 HU (Figure 1). The adjacent celiac trunk and initial segment of the superior mesenteric artery were wrapped by the tumor. Multiple thickened, tortuous vascular shadows were observed at the tumor margins, around which there were multiple enlarged lymph nodes. These enlarged lymph nodes were also grossly enhanced. The main pancreatic duct was markedly dilated. Routine and contrast-enhanced pancreatic MRI demonstrated an irregularly shaped mass in the pancreatic head/uncinate process that was approximately 4.3 × 6.4 cm^2^ in size. T2-weighted imaging (T2WI) revealed nonhomogeneous, slight hyperintensity, whereas T1-weighted imaging (T1WI) revealed hypointensity. Patchy hypointensity on both T1WI and T2WI were observed within the lesion. DWI showed slight hyperintensity. Severe heterogeneous enhancement of the mass was observed on a contrast-enhanced scan (Figure 2), and the mass wrapped around the proximal part of the adjacent superior mesenteric artery. Multiple enlarged lymph nodes were observed around the mass. The possibility of a malignant neuroendocrine neoplasm was considered preoperatively.

**FIGURE 3:**
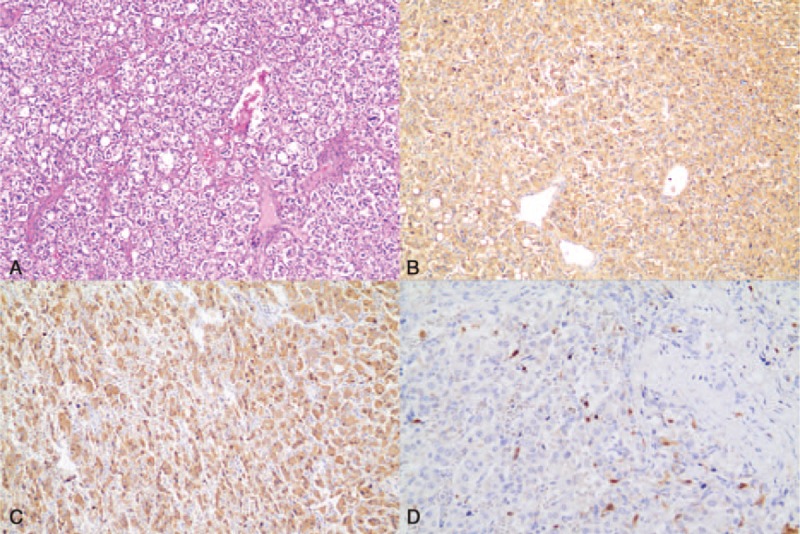
(A) Microscopy shows that the oncocytes are nest shaped and are divided by fibers interspersed with blood vessels. The oncocytes are oval or multiform, the kernels are round, and nuclear atypia is rarely observed (hematoxylin–eosin staining, original magnification ×100). (B) Tumor cells positively stained for CgA (original magnification ×100). (C) Tumor cells positively stained for Syn (original magnification ×100). (D) Tumor cells partially positively stained for S-100 (original magnification ×100).

After preoperative preparation, the patient underwent pancreatoduodenectomy. Intraoperatively, the mass was observed to be located in the pancreatic uncinate process. This mass was approximately 4 × 6 cm^2^ in size, hard in texture, and wrapped around the superior mesenteric artery and vein. Multiple enlarged, hard lymph nodes were noted at the superior border of the pancreas along the mesenteric vessels. The blood supply around the tumor was abnormally rich. Surgical specimens showed that the mass was located in the pancreatic uncinate process and that the stomach, duodenum, pancreas, and bile duct margins were negative. Immunohistochemistry showed CK (pan) (−), Melan-A (−), CgA (+), Syn (+), S-100 (+), Ki-67 (2%), CD56 (+), and β-catenin (membrane +) (Figure 3). The diagnosis was finalized as pancreatic paraganglioma. Because the tumor in our case grew invasively, the biological behavior was suggested to be malignant. No tumor cells were observed within the submitted lymph nodes. After surgery, the patient recovered well and presented no tumor recurrence on ultrasound reexamination 1 month postoperatively.

## DISCUSSION

Adrenal paragangliomas are common paragangliomas that occur in the retroperitoneum. In contrast, extra-adrenal paragangliomas rarely occur and are considered to account for approximately 15% of the total incidence of paragangliomas.^23^ Pancreatic paraganglioma is a very rare disease; to our knowledge, there are fewer than 30 reported cases in the English-language literature.^3–22^ Pancreatic paraganglioma occurs between ages 19 and 85 (mean age: 52 years), and its male-to-female incidence ratio is approximately 1:2. Approximately half of retroperitoneal paraganglioma patients have symptoms associated with excessive catecholamine secretion, including palpitation, headache, and hypertension.^24^ By contrast, pancreatic paragangliomas are usually nonfunctional, with functional cases observed only occasionally.^3–22^ Clinical symptoms of pancreatic paraganglioma lack specificity and include abdominal pain, abdominal discomfort, right flank discomfort, anemia, hypochondralgia and an abdominal mass, and 1/6 to 1/7 cases may be asymptomatic and are discovered by chance.^3–22^ In a few cases, mildly mobile masses with tenderness are palpable on physical examination.^4,17,18^ Among these pancreatic paraganglioma cases, 1 functional pancreatic paraganglioma patient may develop intermittent hypertension, whereas another patient's condition may be complicated by hypertension, diabetes, and hyperlipidemia.^19^ Twenty-four-hour urinary norepinephrine excretion is increased markedly in functional pancreatic paraganglioma cases,^19^ whereas nonfunctional pancreatic paragangliomas may exhibit slightly elevated carbohydrate antigen 19-9 and cancer antigen 15-3 levels.^10,15^ The pancreatic paraganglioma in our case was discovered accidentally; because the possibility of paraganglioma was not considered preoperatively, relevant endocrine indices were not detected. The tumor marker ferritin index was elevated but lacked specificity. Retroperitoneal malignant paraganglioma has a relatively high incidence of approximately 28%.^23^ In comparison, there are only 3 reported cases of malignant pancreatic paragangliomas,^16,17,19^ with an incidence of approximately 15%.

The histopathological feature of paragangliomas is well-defined cell nests (“Zellballen”). Cell nests are mainly composed of chief cells, within which there are many catecholamine-containing granules that are surrounded by a thin layer of sustentacular cells.^24^ Paraganglioma is a hypervascular lesion in which the chief cells are also closely associated with fenestrated capillaries.^1^ Therefore, paragangliomas always appear as hypervascular masses on imaging.

Pancreatic paragangliomas mostly occur in the pancreatic head/uncinate process and rarely occur in the pancreatic body or tail, and their sizes are 1.5 × 1.5 to 17.0 × 19.0 cm^2^.^3–22^ Ultrasound findings are usually hypoechoic masses with a clear or an ill-defined boundary and an abundant blood supply, and there can also be anechoic areas within the masses.^6,11,12,19–22^ A few of the pancreatic paraganglioma cases reported in the last century were mistaken for pancreatic or mesangial cysts when they showed cystic masses on CT examinations.^3–5^ We speculate that the misdiagnosis may have occurred because contrast-enhanced CT was not widely used at that time and routine CT alone was not sufficient for identification of the blood supply of the lesions and their margins. Using intravenous contrast agents for abdominal masses, the imaging evaluation of pancreatic paraganglioma has been enriched. Pancreatic paraganglioma usually presents as an isodense or hypodense mass with a clear or an occasionally ill-defined boundary on routine CT, while showing severe enhancement on contrast-enhanced scans, which may be accompanied by different forms of unenhanced areas.^6,7,9–12,14–22^ Individual cases can demonstrate intratumoral vessels and draining veins.^11,20,22^ Other manifestations include an atrophic pancreatic body and tail and a dilated pancreatic duct.^11,14,21^ Similar to paragangliomas in other regions, pancreatic paraganglioma is usually manifested as hypervascular.^3–22^ Cystic changes and necrosis are common in pancreatic paraganglioma, whereas calcification is rarely observed.^3–22^ The patient in our report had obvious central calcification; previously, there was only 1 case that reported slight calcification.^6^ In contrast to pheochromocytoma, which may be accompanied by intratumoral hemorrhage, no clear intratumoral hemorrhage was observed in any of the reported pancreatic paragangliomas.^3–22^ Previously, there were only 2 cases in which the MRI findings of pancreatic paraganglioma were reported.^7,22^ In contrast to the previous reports, in our case, T1WI showed hypointensity, T2WI showed slight hyperintensity, and DWI showed hyperintensity, which is similar to what is observed for pancreatic carcinoma. The MRI findings of pancreatic paraganglioma lack the typical signs of paragangliomas, such as the “salt-and-pepper” or “light-bulb” sign. However, in our case, similar to what is observed for pheochromocytomas, contrast-enhanced MRI showed severe enhancement in the arterial phase and reduced enhancement in the venous phase, demonstrating a washout pattern. We share the view that a clear display of draining veins may contribute to the diagnosis of pancreatic paragangliomas,^10,22^ which was also confirmed by both contrast-enhanced CT and MRI in our case. The abundant draining veins of pancreatic paragangliomas may be related to the rich blood supply of the tumor itself and the angiogenic factors in the tumor. Despite the view that this imaging sign is present in approximately 50% of pancreatic paragangliomas,^22^ we believe that the incidence and specificity of this imaging characteristic need to be further explored. We are the first to report the DWI sequence signal changes of pancreatic paraganglioma. However, these changes’ value in the diagnosis and differential diagnosis of pancreatic paraganglioma and in the differentiation of benign and malignant paragangliomas needs further exploration.

In 2012, Higa and Kapur^16^ reported the first case of malignant pancreatic paraganglioma. However, due to the lack of specific pathological characteristics, no consensus has been reached on diagnostic criteria for malignant paraganglioma. Low reactivity for neuropeptides and high division are considered to be associated with malignant paraganglioma, whereas the presence of pleomorphism, mitosis, and vascular invasion is not necessarily related to the poor clinical course and prognosis.^16^ Kimura et al^25^ attempted to evaluate the biological characteristics of paraganglioma using a scoring system; however, in the well-differentiated and moderately differentiated categories, 13% and 63% cases, respectively, still presented with malignancy. Currently, lymph node metastasis and metastases in other organs are still definite evidence for the confirmation of malignant paraganglioma.^26^ Among the previously reported 3 cases of malignant pancreatic paragangliomas, 2 cases showed only 1 lymph node metastasis, whereas the remaining case presented with multiple intrahepatic metastases.^16,17,19^ In the case report by Al-Jiffry et al,^17^ CT failed to detect any enlarged lymph nodes, despite the pathological confirmation of lymph node metastasis. By contrast, in our case, markedly enlarged lymph nodes were detected on both CT and MRI, suggesting lymph node metastasis, but the histopathology findings were negative. Therefore, caution is needed in the imaging evaluation of lymph nodes for pancreatic paragangliomas due to the presence of false negatives and false positives. Nevertheless, CT can detect the metastases of malignant paragangliomas in other organs as well as tumor recurrence.^17,19^

Surgical resection, including simple mass resection and pancreaticoduodenectomy, is the preferred treatment for pancreatic paraganglioma.^17,21^ For functional pancreatic paragangliomas, the preoperative administration of α-adrenergic receptor blockers contributes to successful surgical procedures.^19^ One patient with functional pancreatic paraganglioma lost the chance for surgical resection due to intraoperative hypertension.^19^ Most described pancreatic paraganglioma patients had good surgical outcomes and were alive during 14-month to 5-year follow-ups.^6,7,9,12,14,16–19,22^ Two pancreatic paraganglioma patients died, 1 of whom was a patient with malignant pancreatic paraganglioma and multiple liver metastases who had received chemotherapy and lived for 4 years.^8,19^ In our case, although the patient had peripheral tumor invasion, no recurrence or metastasis was found on short-term reexamination.

## CONCLUSIONS

Paraganglioma of the pancreas is a rare neoplasm. Most pancreatic paragangliomas are nonfunctional paragangliomas that occur in people in their 50s and more frequently in women; pancreatic paragangliomas also often lack specific clinical symptoms. Most tumors are located in the pancreatic head. Imaging findings include hypervascular masses with draining veins on contrast-enhanced CT or MRI, often accompanied by necrosis or cystic changes and occasionally by calcification. Surgical resection is the preferred treatment for pancreatic paraganglioma, which has a good prognosis.
